# Early motherhood: voices from female adolescents in the Hohoe Municipality, Ghana—a qualitative study utilizing Schlossberg’s Transition Theory

**DOI:** 10.1080/17482631.2020.1716620

**Published:** 2020-01-24

**Authors:** Sitsofe Gbogbo

**Affiliations:** School of Public Health, University of Health and Allied Sciences, Ho, Ghana; Institute of Education, Johannes Gutenberg University Mainz, Germany

**Keywords:** Female adolescents, adolescent mothers, early motherhood, qualitative research, Schlossberg’s Transition Theory

## Abstract

**Purpose**: Using Schlossberg’s Transition Theory, this study explored the lived experiences of pregnant adolescents and adolescent mothers coping strategies during their transition to motherhood.

**Methods**: Based on a phenomenological perspective, this qualitative study used in-depth interviews (IDIs) and focus group discussions (FGDs) to answer the research aim. The process of data gathering included 8 FGDs and 12 IDIs held with adolescent mothers and pregnant adolescents. Audio recordings were transcribed and imported into MAXQDA 2018 for analysis.

**Results**: Applying the interpretative phenomenologial analysis (IPA), four key themes emerged. This included: news of pregnancy; adolescent motherhood; social support and coping strategies. The findings display female adolescents’ expressions of some of the negative aspects of motherhood they have been confronted with that have affected their lives. The dominant societal challenges posited by the adolescents included coping with economic and financial constraints, opting for unsafe abortion to reduce stigma associated with unplanned pregnancy, and managing the extra responsibility of taking care of the baby and the challenge of going back to school after delivery.

**Conclusion**: There is the need to build social capital among community members regarding their support to improve the psychosocial well-being of adolescents during early motherhood.

## Introduction

Around 16 million girls aged between 15 and 19 and 2.5 million girls below 16 years give birth each year globally (Blum & Gates, 2015). Additionally, more than 90% of adolescent pregnancies occur in low- and middle-income countries (LMICs) (Nove, Matthews, Neal, & Camacho, 2014). Adolescence, defined as the age between 10 and 19 years, marks a transition of life from childhood to adulthood (Sawyer, Azzopardi, Wickremarathne, & Patton, 2018) and is considered a critical stage in the life of an individual (Kar, Choudhury, & Singh, 2015). During this period, due to hormonal changes, adolescents may feel they are matured enough to engage in sexual debut (Atuyambe, Mirembe, Johansson, Kirumira, & Faxelid, 2005).

Adolescent childbearing is a major public health issue (Ngum Chi Watts, Liamputtong, & McMichael, 2015) and has received considerable attention in the past years, due to the extensive range of health-related problems associated with it (Islam, Islam, Hasan, & Hossain, 2017; Roberts, Graham, & Barter-Godfrey, 2011). Adolescent pregnancy is one contributing factor to maternal and child mortality across many countries (Nove et al., 2014), and also in the cycle of intergenerational poverty and poor health (Sawyer et al., 2018). Evidence has shown that there is an association between adolescent pregnancy and socio-economic difficulty, which includes discontinuation of education, single parenting, and reduced employment prospects (McMichael, 2013). Adolescent mothers are at a high risk of experiencing puerperal endometritis, eclampsia, systemic infections, preterm delivery, low birthweight, and severe new-born illnesses than their older counterparts (Ganchimeg et al., 2014).

The majority of adolescent pregnancies and births are unplanned (Sedgh, Finer, Bankole, Eilers, & Singh, 2015), often resulting in affected adolescents being stigmatized (Maly et al., 2017). This can have many negative social and economic effects on the girls, their families, and communities as a whole. Furthermore, most girls who get pregnant before the age of 20 years struggle to adjust to their new roles as mothers (Ngum Chi Watts et al., 2015). Adolescents are emotionally and psychologically not prepared for motherhood and, thus, experience a high rate of prenatal and postnatal depression (Hodgkinson, Beers, Southammakosane, & Lewin, 2014).

Sub-Saharan Africa (SSA) has the highest adolescent fertility rate globally, yet records the lowest rates of contraceptive prevalence use (WHO Regional Office for Africa, 2012; Yakubu & Salisu, 2018). Female adolescents living in rural areas have a higher fertility rate and this presents both health and social consequences (Ghana Health Service, 2017). A survey conducted in Ghana in 2017 indicated that 14% of 15- to 19-year-old female adolescents had experienced pregnancy; of these, 12% had already had a live birth and 3% were pregnant with their first baby (Ghana Statistical Service & Ministry of Health, 2018).

Motherhood is an essential part of the social and cultural system in Ghana. Mothers occupy positions in society based on the relationship they have with a child (Waterhouse, Hill, & Hinde, 2017). As revealed by Rosato and colleagues, women’s voices need to be heard by decision and policymakers (Rosato et al., 2006). Listening to the lived experiences of pregnant adolescents and adolescent mothers and how they cope with their challenges during early motherhood will facilitate the improvement of adolescent-friendly health services and also promote culturally sensitive, quality health care (Wilkinson & Callister, 2010).

Research has shown that studies done on transition have mostly focused on the support given to individuals struggling with events such as loss of employment, loss of relatives, and other serious ordeals (Anderson, Goodman, & Schlossberg, 2012). Interventions such as counselling using the 4 S’s framework of Schlossberg’s Transition Theory were designed to assist individuals who are struggling to adjust to major changes in their lives (Browne, Webb, & Bullock, 2018).

This study builds on previous research conducted on adolescent pregnancy, unsafe abortion, and the use of contraceptives in Ghana (Agyei, Biritwum, Ashitey, & Hill, 2000; Awusabo-Asare, Abane, & Kumi-Kyereme, 2004; Gyesaw & Ankomah, 2013). Another study has also explored the experiences of adolescent motherhood (Krugu, Mevissen, Münkel, & Ruiter, 2016). In this study, Schlossberg’s Transition Theory (Anderson et al., 2012; Evans, Forney, Guido, Patton, & Renn, 2010) is used as a guide to provide more insight into the lived experiences of pregnant adolescents and adolescent mothers in the Hohoe Municipality of Ghana. Through this theoretical framework, the female adolescents’ perceptions of their own transitions into, through and out of early childbearing are analysed. The research question for this study is, how do female adolescents cope with adolescent pregnancy and what are their views of experiencing early motherhood? This study aimed to explore and describe the lived experiences of pregnant adolescents and adolescent mothers and the strategies they utilized during their transition to early motherhood.

## Theoretical framework

To explore the underlying research question, the study drew on Schlossberg’s Transition Theory, which is usually classified as a theory of adult development (Evans et al., 2010). The theory allows researchers to better appreciate what individuals experience during a transition period (Anderson et al., 2012; Byrd, 2017). The theory defines transition as any event or non-event in which the outcome transforms relationships, practices, expectations, and responsibilities (Goodman, Schlossberg, & Anderson, 2006). Changes may occur in a life situation but if the person involved does not attach much importance to the event, such changes cannot be considered as a transition. For the female adolescents who participated in this research, the above characteristic was a central part of their experiences. The participants, while narrating their lived experiences, attached much significance to the situation in which they found themselves.

To better comprehend the influence a transition has on an individual, Anderson and colleagues explain that it is necessary to consider the type, context, and impact of the transition (Anderson et al., 2012). The three types of transitions described are anticipated transitions (expected events), unanticipated transitions (unexpected events), and non-events (events that are expected to occur but do not occur) (Evans et al., 2010; Goodman et al., 2006). The context of the transition refers to the individual’s relationship to the transition and the settings in which the transition takes place. The impact is determined by how the transition influences the day-to-day life of the individual (Anderson et al., 2012; Evans et al., 2010; Goodman et al., 2006). In this study the unanticipated transition will be considered, as the adolescents in this study did not plan their pregnancy but got pregnant unexpectedly.

Although a transition may be triggered by an event or non-event, dealing with a transition is a process that increases over time, involving a series of stages, namely “moving in, moving through, and moving out” (Evans et al., 2010). There are four main sets of elements that influence an individual’s capability to cope during a transition period. These are situation, self, support, and strategies, which are also known as the 4 S’s (Goodman et al., 2006). Coping effectively during a transition depends on the individual’s assets in the four sets of elements. The individual’s assessment of the transition period is a significant factor in the coping process. It is worth noting that the 4 S’s provide an outline for an individual’s assessment process (Evans et al., 2010). Consequently, the 4 S’s described by Schlossberg’s Transition Theory can be utilized to answer the questions of the situation in which the adolescent women in this study found themselves, the social support available to them, how their personal and demographic characteristics affect, how they view life, and how they cope with early motherhood. While the literature has revealed the effectiveness of Schlossberg’s theory in practice, research studies backing its rationality are insufficient (Evans et al., 2010). However, this study proposes that as a theoretical framework, it captures participants’ own perceptions of their experiences in early motherhood and how they cope with their transition.

## Materials and methods

### Study location

The study was conducted in the Hohoe Municipality, one of the 25 administrative districts in the Volta Region of Ghana. According to the 2010 Population and Housing Census, Hohoe Municipality represents 7.9% of the total population in the Volta Region with a population of 167,016. It includes 52.1% females and 47.9% males. The population of the municipality is mainly young, with children under the age of 15 representing 35.9% (Ghana Statistical Service, 2014). Trends in adolescent pregnancies by region in 2014 to 2016 show that the Volta Region has the second highest rate of adolescent pregnancy with a record of 10,296 adolescent pregnancies on record, representing 15.3% of the total number of recorded teenage pregnancies in Ghana (Ministry of Health, 2016). This indicates that adolescent pregnancy is still high in the region and this needs to be tackled.

The municipality covers an area of 1,403 sq.km and is divided into seven districts, namely, Hohoe, Alavanyo, Agumatsa, Gbi-Rural, Likpe, Lolobi, and Akpafu/Santrokofi. The municipality is bounded on the north by Jasikan District, north-west by Biakoye District, west and south-west by Kpando Municipality, south by Afadjato South District, and east by the Republic of Togo (Tarkang et al., 2017). The municipality has a total of 21 health facilities which is made up of 1 hospital located at Hohoe (the District capital town and the only referral facility that provides secondary and tertiary care), 14 health centres, and 6 Community-Based Health Planning and Services (CHPS) compounds (Solomon et al., 2017).

### Study design

This qualitative study used interpretative phenomenological analysis (IPA). In qualitative studies, the researcher endeavours to develop an understanding of the phenomena under study, and to be more perceptive of the various narrations individuals make of their experiences, in order to make more meaning (Creswell, 2007). The use of phenomenology was because it has been considerably beneficial in health sciences and is considered the best applicable method when exploring people’s lived experiences or phenomena that are socially intricate (Creswell, 2007; McMichael, 2013; Ngum Chi Watts et al., 2015).

The study is embedded in IPA because it has theoretical foundations in phenomenology, hermeneutics, and idiography as explained by Pietkiewicz and Smith (2012); Smith (2011). IPA is phenomenological because it utilizes a person’s subjective account rather than the construction of objective descriptions (Smith, Flowers, & Larkin, 2009). Analysing someone else’s experience can be complex; it entails a process of engagement and interpretation by the researcher and this links IPA to a hermeneutic perspective (Smith, 2011). Also, IPA is idiographic, as it requires an analysis of each narration in a corpus in detail, representing in-depth assessment of the lived experience of each participant followed by the search for patterns throughout each narration (Smith & Osborn, 2015).

IPA has been put forward as an appropriate method for exploring how individuals make meaning of their personal and social world; it has been designed as a unique method to conduct qualitative research which offers a theoretical basis and a comprehensive practical guide for conducting qualitative analysis (Smith & Osborn, 2015). The primary aim of IPA is to explore in detail the process of understanding a specific phenomenon in the lifeworld by focusing on how individuals make sense of their experiences (Smith & Osborn, 2015). As the aim of this study is to explore the lived experiences of adolescent pregnant girls and adolescent mothers and examine how they perceive their own behaviour, the researcher considered IPA to be the suitable instrument for analysis. This approach allowed the researcher to discover some aspects of the lives of these adolescent women that would not have emerged during casual conversation (Omari, Wynaden, Al-Omari, & Khatatbeh, 2017).

### Participants

Purposive and snowball sampling methods were utilized to identify this difficult-to-reach population (Creswell, 2007) within the seven districts in the Hohoe Municipality. Initially, the researcher posted flyers about the study on notice boards in various health facilities and churches. Potential participants were asked to contact the researcher through a phone call if they were interested in participating in the study. This method was not successful, as only five pregnant adolescents and five adolescent mothers contacted the researcher and were willing to participate in the study after it had been explained to them. Hence, snowball sampling methods were eventually used in this study to recruit potential participants who met the eligibility criteria (Berg, 2001) of being an adolescent (10–19), pregnant or a mother with a child below the age of 2 years, residing in the Hohoe Municipality and able to speak the Ewe language. The researcher contacted gatekeepers (Chiefs, Queen-mothers, Assemblymen, Traditional Birth Attendants, and Midwives) in the seven districts, to help identify and recruit potential participants. Community members, parents, and potential participants who heard about the study and were interested referred other potential participants (Creswell, 2007). In total, 100 participants were identified and invited, of which eight adolescent mothers did not meet the eligibility criteria because their children were above 2 years of age. Forty-six pregnant adolescents and 46 adolescent mothers with a mean age of 17 years (ranging from 15 to 19 years) met the eligibility criteria. Appointments were arranged with eligible participants who agreed to participate in the study at a time and venue suitable to them.

### Respondents’ characteristics

In this study, a total of 92 adolescent mothers and pregnant adolescents, aged 15 to 19 years, were interviewed and participated in FGDs. Twelve participated in the IDIs and 80 participated in the FGDs, out of which 10 mothers were back in school after initially dropping out. Out of the 92 participants, 15 were undergoing skills training. All participants were unemployed, except two who were engaged as salespeople in two shops. Most of the participants were either living with their parents or grandmothers or with the father of their baby. Of the 92 participants, 15 were from Hohoe, 25 from Alavanyo, 10 from Agumatsa, 10 from Gbi-Rural, 20 from Likpe, 7 from Lolobi, and 5 from Akpafu/Santrokofi. All participants were either Christians or Muslims (85 Christians and 7 Muslims) and spoke at least two languages. As shown in Table I.

**Table 1.** Respondents’ characteristics

Participant characteristics	IDI (description and number)	FGD (description and number)
Age range	15–19 years	15–19 years
Pregnant adolescent	6	40
Adolescent mother	6	40
Educational status	Primary school dropout (2) JHS dropout (6) Completed JHS (4)	Primary school dropout (15) JHS dropout (54) Completed JHS (11)
In-school after initial dropout	3	7
Marital status	Married (1) Unmarried (11)	Married (3) Unmarried (77)
Employment status	Employed (0) Unemployed (12)	Employed (2) Unemployed (78)
Dwelling place	Hohoe (2) Alavanyo (2) Agumatsa (2) Gbi-Rural (2) Likpe (1) Lolobi (2) Akpafu/Santrokofi (1)	Hohoe (13) Alavanyo (23) Agumatsa (8) Gbi-Rural (8) Likpe (19) Lolobi (5) Akpafu/Santrokofi (4)
Religion	Christian (10) Muslim (2)	Christian (75) Muslim (5)
Total Participants	12	80

### Data collection tool and procedures

Data applied in this study was collected between August and October 2017 as part of a larger qualitative research project that explored adolescent transition to parenthood in the Ghanaian context. Data for this study was collected applying the principles of IPA using in-depth interviews (IDIs) and focus group discussions (FGDs). IDIs and FGDs are known methods applied in IPA studies. IDIs were conducted with 12 participants (6 pregnant adolescents and 6 adolescent mothers) while 8 FGDs were conducted with 80 participants (40 pregnant adolescents and 40 adolescent mothers). The interview guide was pilot-tested by the researcher and two research assistants on two pregnant adolescents and two adolescent mothers in one nearby district to the study setting. Pilot-testing the interview guide helped the researcher to refine the interview questions and also familiarize herself with the research method (Flick, 2014). Interview topics for both IDIs and FGDs included coping with pregnancy and motherhood as an adolescent, economic and financial restrictions, and social, emotional, and psychological demands. Interviews were conducted by the researcher and two research assistants in Ewe, the main language spoken in the study area, and translated into English. All IDIs were conducted in participants’ home at their own convenience and all FGDs were conducted in private and convenient environments in three of the districts. To maintain confidentiality, pseudonyms were assigned to all participants (Berg, 2001). Interviews and discussions were digitally recorded, with permission from participants, and transcribed verbatim. IDIs lasted between 40 and 60 min while FGDs lasted between 60 and 90 min.

### Data analysis

To describe the experiences of teenage pregnancy and early motherhood among adolescent women, the researcher analysed the data using an iterative and inductive approach and followed the six steps outlined in Smith et al.’s (2009) IPA framework: (1) reading and re-reading; (2) initial noting; (3) developing emergent themes; (4) searching for connections across emergent themes; (5) moving to the next case; and (6) looking for patterns across cases. Each step was done by the author and is described below. Transcripts were first analysed individually, then compared for similarities and differences.

First, the researcher read and re-read the transcript of the first participant without coding while listening to the audio-recording, in order to immerse herself in the data. After the transcript has been read a few times, the researcher moved to the next step of initial noting; by examining the transcript line by line while making notes of anything that was of interest, to have a better understanding of how the participant experienced pregnancy and early motherhood. At this stage, three core categories were developed; descriptive comments focused on unfolding what the participant said (shown as normal sentence in Table II), linguistic comments focused on exploring the participant’s exact use of language (shown as italic sentence in Table II), and conceptual comments focused on engaging at further probing and theoretical level (shown as underlined sentence in Table II) (Smith et al., 2009); thus, the researcher developed potential connection within the transcript.

**Table 2.** Initial noting and developing emergent themes from one participant

Emergent themes	Original transcript	Initial notes
Age of getting pregnant Feeling neglected Negative thoughts due to neglect Not experienced with signs of pregnancy Feeling of sadness Regret and disappointment from mother Emotional support from grandmother Reassurance from grandmother as a form coping strategy	I: Please are you okay to tell me something about yourself P: Hmmm I am just emm 15yrs … … 15 yrs old and I was living with my parents ooo but now living with my grandmother since I got pregnant. Um … I was impregnated 6 months ago by myyyy boyfriend who is 18yrs old and … … … he refused to accept the pregnancy because he said I have other boyfriends and that he is not responsible for it. I wanted I wanted to kill myself because I felt so rejected. I: How did you feel when you realized that you were pregnant, how did you cope with your emotions. P: Hmm initially, I didn’t know that I was pregnant, em em I was still having my period until 4 months when I started vomiting and sleeping always in class. My teacher called me and asked me if I was pregnant, I said no and then she asked me if I have missed my period and I said no. One day my mother said she was taking me to the clinic for malaria test but emm, when we got to the clinic, she asked the nurse to do a pregnancy test on me and the result was positive. I started crying and my mother started beating me, she said I have brought disgrace to her and that she worked hard to send me to school and I pay her back with pregnancy. When we got home that day I run and went to my grandmother because my mother said she will beat me till I die. So, I am still staying with my grandmother, she treats me with lots of love and care. Her encouragement helps me to cope with the disappointment, she is really a source of emotional support for me.	*Was she finding it difficult to mention her age? The use of hmmm and repeating her age sounded like she might not be comfortable that she got pregnant at age 15*. A young girl impregnated by another teenager who refuses to accept responsibility of pregnancy. *Emphasizing the possibility of killing herself by the repetition of the word “I wanted”* Major issues of denial and negative thinking of self, which may result in suicide. Major issue of unawareness of pregnancy, so she didn’t know of her pregnancy probably because she didn’t miss her period? Ignorant of being pregnant in early stages She was sad to know she was pregnant and probably not expecting the news of pregnancy. Parent showing disappointment in her daughter’s pregnancy This led her to ran away from home. Showing signs of rejection. Preferred staying with grandmother because of the love and support shown her by her grandmother. She realized her grandmother accepted her regardless of her circumstance.

In step three, the researcher developed emergent themes from the initial notes. The researcher tried to lessen the volume of the transcript and initial notes, by mapping the interrelationships, connections, and patterns between the initial notes and grouped them into somewhat broader emergent themes. This involved a critical move to working mainly with the initial notes rather than the transcript itself. Steps two and three are illustrated below in Table II, containing a short extract from an interview with one of the pregnant teenagers who talked about herself and her experiences of how she felt, when she realized, she was pregnant.

In the fourth step the researcher searched for connections across the emergent themes by organizing the emergent themes into broader categories of super-ordinate themes that represent the participant’s experiences and her interpretation of motherhood, while incorporating elements of phenomenological knowledge that aimed to keep the meaning of that piece of narration at an advanced level of abstraction (Chapman, Parameshwar, & Jenkins, 2007). Step four is illustrated below in Table III.

**Table 3.** Super-ordinate themes and themes from one participant

Super-ordinate themes	Themes	Subthemes
News of pregnancy	Feeling of sadness Feeling neglected Negative thought Regret and disappointment from mother	Started crying He refused to accept the pregnancy He said he is not responsible for the pregnancy I wanted to kill myself I felt rejected She said I have brought disgrace to her She said she will beat me till I die
Adolescent motherhood	Age of getting pregnant	I am just 15yrs I was impregnated 6 months ago My boyfriend who is also 18yrs old
	Not experienced with signs of pregnancy	I didn’t know I was pregnant She asked me if I have missed my period and I said no
Social support	Emotional support	She treats me with lots of love She is really a source of emotional support
Coping Strategy	Reassurance from grandmother	Her encouragement helps me to cope with the disappointment

In step five, the researcher moved to the next participant’s transcript and repeating the process for all the other participants. As such, each participant’s transcript was individually read and re-read, after which the researcher moved to the next step of making initial descriptive, linguistic, and conceptual comments. The researcher then examined these notes while grouping them into emergent themes. The researcher finally searched for connections across the emergent themes and then categorized them under super-ordinate themes.

In the final stage of the analysis, of looking for patterns across cases, the researcher compiled all the themes from the transcripts, looking for connections and clusters to assess key themes. At this stage, because of the large number of participants in this study, the researcher also measured recurrence across each text, which is considered a way of enhancing the validity of the findings (Smith et al., 2009). With the aid of MAXQDA 2018 (qualitative data analysis software), the relationship between themes and exact narrations of participants was clarified. Four key themes emerged from the analysis, which were news of pregnancy, adolescent motherhood, social support, and coping strategies.

### Ethical approval

The study was approved by the Ghana Health Service Ethics Review Committee (Ethical approval ID No. GHS-ERC: 006/07/17) and conducted in accordance with the Helsinki Declaration. Anonymity, voluntary participation, and written informed consent were significant requirements (Carlson, Boyd, & Webb, 2004) for participation. Potential participants were given detailed information about the study and a consent form to sign after they had agreed to participate in the study. Participants who were below the age of 18 years had their parent’s or guardian’s consent to participate in the study.

No individual was coerced, induced, or deceived to participate in the study and was given the opportunity to express their feelings and experiences about early pregnancy and motherhood. All respondents signed/thump printed a consent form to show their willingness to participate. Individuals had the right to withdraw from the study at any time.

## Results

The analysis of the lived experiences of pregnant adolescents and adolescent mothers and the strategies they utilized during their transition to early motherhood resulted in four key themes; news of pregnancy, adolescent motherhood, social support, and coping strategies. Overall, the four themes that emerged from this study depict pregnant adolescents and adolescent mothers shared and lived experiences transitioning into motherhood. The key themes reflect their experiences, what social support systems exist for adolescents and what coping strategies adolescents explore during their transition into early motherhood.

### Theme I: news of pregnancy

Getting pregnant as an adolescent was a difficult experience for some of the participants; their parents were disappointed, also realizing that they were pregnant made them dislike themselves, because the pregnancy was not planned. Some respondents who recounted their experiences after been confirmed to be pregnant reported they attempted to abort the pregnancy out of disappointment as captured in the words of one adolescent mother.

when I got pregnant, my father was very disappointed in me and that made me hate myself for getting pregnant; I even tried aborting the pregnancy. (17-year-old adolescent mother)

Expressions such as our parents’ inability to take good care of us and we are from poor socio-economic backgrounds were mostly cited in FGDs and IDIs as reasons that led to them becoming pregnant unexpectedly. Participants avowed they and their parents however were devastated after the news of pregnancy.

My parents did not have the money to provide my basic needs, so I went for a boyfriend and I got pregnant the first time we had sex. (18-year-old pregnant adolescent girl)

Beneath this sentiment of parental neglect, adolescents also reported they felt very disheartened and regretful at getting pregnant at an early age. There were participants who were unhappy about their situation and felt embarrassed about their conditions as espoused by one adolescent mother.

I was very disappointed because that was the first time, I had sex …………. I wasn’t myself for months. (17-year-old adolescent mother)

One dominant expression by pregnant adolescents and adolescent mothers was the “act of denial” by their boyfriends about the pregnancy as one pregnant adolescent recount her experiences.

He refused to accept the pregnancy because he said I have other boyfriends and that he is not responsible for it. (15-year-old pregnant adolescent girl)

Other dominant views expressed by adolescents included suicidal thoughts after pregnancy confirmation and the feeling of rejection by family and friends. Adolescents who reported a rejection by family and friends reported they felt stigmatized both in their families and in the community as a whole.

I wanted to kill myself when I realised, I was pregnant, I felt very excluded and criticised. (18-year-old pregnant adolescent girl)

### Theme II: adolescent motherhood

Early motherhood brought mixed feelings and moments of depression as adolescents recount. Early motherhood for adolescents brings a lot of challenges and anxiety. Adolescent’s lack of self-worth and confidence and their lack of knowledge on how to care properly for their new-borns was also cited as one major challenge associated with early motherhood as one adolescent mother avowed.

I don’t think I am ready to be a mother as a teenager; I am not confident enough; sometimes, I struggle to take good care of my baby, I get very irritated and worried. (16-year-old adolescent mother)

Shared experiences during FGDs and IDIs among participants revealed their experiences after becoming adolescent mothers can be described as negative. Mothers reported that the lack of support from their families was one reason that they felt they had negative experiences as early mothers. One adolescent mother whose experience was similar to some other mothers narrates how early motherhood was a challenge for her given the absence of parental and family support.

It’s hard time for me as a mother, I have no one to go to, my parents are dead …………… I will work hard to make my son a better person. (19-year-old adolescent mother)

Furthermore, some participants reported that if challenges such as support from family are resolved it would motivate them to go back to school.

If my mother agrees to take care of my baby I will go back to school. (16-year-old adolescent mother)

Beyond the reality and experiences of early motherhood narrated by adolescents was their deep commitments to pursue their educational goals and attain an economic status that will enable them take good care of their children. Going back to school was perceived as a good option so that they will give confidence to their children when they grow up to know that their mothers were able to achieve higher education although they went into motherhood early.

I am determined to go back to school, this will open better opportunity for me to bring up my child well even though I became a mother early. (17-year-old adolescent mother)

Adolescents however acknowledged some challenges such as their inability to raise funds to pay for their education and the lack of employment opportunities for them to earn some income as early mothers. Given limited employment opportunities, learning a trade was a commonest option for some adolescent mothers as captured by one adolescent mother.

I would like to learn tailoring; I wish to go back to school but I don’t have anybody to pay my fees and take care of my child. But if I learn the tailoring, I will be able to take care of myself and my son. (19-year-old adolescent mother)

### Theme III: social support

For some of the participants, even though early motherhood was associated with stigma, they still had some form of support during their transition. Narrating all their experiences of coping with teenage pregnancy and early motherhood, some of them were more enthusiastic about the social support they received despite the challenges they faced. The participants frequently specified that their support came from three sources, which are discussed as sub-themes (support from the adolescents’ family, support from the father of their babies, and support from friends and community members) and were perceived to have a great influence on their determination. Some of the adolescent women stated in the study that the support they received was very important because it helped them build their self-confidence, motivating them to go back to school or commence trading. There were participants who narrated how their families supported them financially, emotionally, and psychologically. Some also narrated how they received support from the father of their baby. Furthermore, two participants recounted the support they received from friends and the community as a whole.

### Support from the adolescent family

In spite of parents getting angry and showing a lot of disappointment in their daughters, some parents were still willing to take care of their daughters. Participants indicated that their parents were a source of support to them and that brought them joy. Even when they felt all was lost, their parents were a great source of motivation for them not to give up in life. For instance, there were participants who stated that their parents expressed interest and were willing to let them go back to school as narrated by one adolescent.

My father wants me to go back to school so, when my baby grows up a little bit, I will give her to my mother and go back to school. (17-year-old adolescent mother)

Mothers and grandmothers were seen to support their daughters and granddaughters no matter how angry and disappointed they were. Some of the participants narrated that their mothers were always there for them, providing most of their needs out of the love that they had for them. They encouraged the girls not to give up but to accept the situation and move on with their life.

My grandmother also helped me to overcome my emotions. She told me everything will be fine after I deliver my baby, so I should not think too much or worry, because it has already happened and there is nothing, I can do about it. (17-year-old pregnant adolescent girl)

### Support from the father of a baby

Some of the participants narrated that they received support from the father of their baby, but this support was mostly insufficient. However, some of the fathers were not supportive because they were students and under the care of their own parents. Others were also not doing any meaningful work that would enable them to take full responsibility.

The father of my baby gives me money sometimes but that is not enough to take care of us, this is because he is a student, he also depends on his parents. (17-year-old adolescent mother)

Some of the adolescent women indicated that they had ended their relationship with their boyfriend before they realized they were pregnant, which made it difficult to approach them for support.

It is difficult to ask him for money because I broke up with him before I realised, I was pregnant. (17-year-old pregnant adolescent girl)

### Support from friends and the community

In the Hohoe Municipality, adolescent pregnancy is frowned upon. There is the perception that it brings shame to the community and does not set a good example for the younger generation. Adolescent girls who get pregnant are seen as a bad influence on their peers. For this reason, they are not given much support, even by their friends, causing them to feel stigmatized. Some of the participants stated that their friends teased them and called them names. The friends whom they could rely on were mostly also in the same situation—either pregnant or also an adolescent mother.

I have a friend just around the corner whom I visit. She is also a teenage mother, so we have a lot of things we talk about; we also share our problems and encourage each other. (17-year-old adolescent mother)

### Theme IV: coping strategies

There were numerous problems connected with early motherhood for some of the participants. One of the biggest challenges they faced during this transition period was financial limitations. In this research, all the adolescent women narrated how they coped with difficult situations. They did not feel financially secure, and some tried to cope by making personal resolutions, missing hospital appointments, and abandoning their babies to avoid embarrassment. Missing antenatal care (ANC) and postnatal care (PNC) is undesirable for both mother and child, as this may have health implications. Some of the adolescent women narrated how worried they felt for not attending ANC due to lack of money for transportation. Yet, they perceived that was what they could do to manage their financial constraints.

I face a lot a lot of money problems, the boy who impregnated me has abandoned me. Even to go for ANC is a big problem for me because my parents say the money, they have they will use to take care of my younger siblings. So, I don’t go and that is how I cope with my money problems. (15-year-old pregnant adolescent girl)

Some of the pregnant adolescents indicated that they intend to abandon their babies at the hospital or leave them at an orphanage after birth because they were not able to cope with the financial pressures that came with getting pregnant as an adolescent, where the father of the unborn child had denied the pregnancy.

I am thinking of leaving my baby at the hospital after I have given birth, I am sure the nurses will get someone to take good care of my baby or they will take the baby to an orphanage. (15-year-old pregnant adolescent girl)

## Discussion

The theoretical framework used in this study was Schlossberg’s Transition Theory, specifically, the 4 S’s framework (Evans et al., 2010). Schlossberg’s Transition Theory addresses transitions in life and how individuals cope with these changes. The 4-S’s of transition comprises Situation: What is happening?; the Self: To whom is it happening?; Support: What help is available?; and Strategies: How does the person cope? (Moran, 2017, p. 101). During the adolescent women’s transition to motherhood, they were faced with these 4 S’s of transition (Anderson et al., 2012). For the purpose of this study, participants narrated their experiences as teenage women who were transitioning from adolescence into motherhood, what support was available to them and what strategies they used to cope with their transition period.

It was clear in the findings that the adolescent women were unhappy about the situation in which they found themselves. Their situation was triggered by an unplanned, badly timed pregnancy, occurring as it did while they were still students (Mjwara & Maharaj, 2018; Timaeus & Moultrie, 2015). The adolescent women in this study mostly did not intend to get pregnant, and therefore were not ready to become mothers when they realized they that were pregnant. Not all these adolescent women were able or willing to make a positive transition to a motherhood identity (Mangino, 2008). Some of the adolescent women in this study narrated how they tried to abort the pregnancy to avoid any embarrassment. This finding is consistent with others (Atuyambe et al., 2005; Ramakuela, Lebese, Maputle, & Mulaudzi, 2016).

Becoming a mother as an adolescent was a challenging time for all the participants in our study. Most of them desired to go back to school or learn a trade in order to give their children a good future. However, they felt they were not in control of their lives and depended on others to enable them to pursue their goals. Having a strong desire to prove themselves as good mothers was also documented by (Macutkiewicz & MacBeth, 2017; Mcdermott, Graham, & Hamilton, 2004) reviews. Empirical evidence by Ntinda, Thwala, and Dlamini (2016) and Chohan and Langa (2011) revealed that adolescent mothers are mostly determined to add value to themselves but are sometimes constrained by various challenges that require many compromises. When providing support to pregnant adolescents and adolescent mothers, it is important to tailor advice and support to their specific circumstances, such as their past experiences, ideas, and expectations regarding the role of motherhood. As discussed by Anderson et al. (2012), using the 4 S’s framework will enable these adolescent women to tackle their challenges with positive expectations, thereby coping successfully with their situation.

Schlossberg’s Transition Theory 4 S’s framework illustrates that individuals who receive support during their transition adapt better to their new situation (Anderson et al., 2012). According to McLeish and Redshaw (2017), support is an essential element during pregnancy and motherhood because women seek the encouragement of people around them. As in this study, Ngum Chi Watts et al., 2015 found that even though adolescent pregnancy and early motherhood were associated with a level of struggle and disappointment, the adolescent women received some form of support from their families, baby’s father, friends, and community. The extent of social support available to the participants in this study depended on how their pregnancy affected other relationships. Most of the people around these adolescent women were disappointed about their pregnancies; parents felt their daughter had brought shame to them. However, because of the love they, especially mothers and grandmothers, had for their daughter, they willingly supported her emotionally, psychologically, and financially. Contrary to the findings in this study, David, Van Dyk, and Ashipala (2017 revealed in their study that adolescent mothers had poor social support from their families.

Finally, participants in this study narrated that they were struggling financially to cope during their pregnancy and early motherhood. Also, due to the stigma that is associated with adolescent pregnancy, participants were overwhelmed by the burden of being a mother. This finding is equally consistent with other studies on adolescents in developed (Ngum Chi Watts et al., 2015; Roberts et al., 2011) and developing countries such as a study among adolescent women in Mulago, Uganda. Their studies suggest that many pregnant adolescent girls struggle to cope with early motherhood and wish they had aborted the pregnancy (Kaye, 2008). As defined by Labrague et al. (2017), coping strategies are behaviours that individuals use to avoid, relieve, or react to a traumatic situation. Results in this study revealed that participants employed a more emotion-focused strategy (Baker & Berenbaum, 2007) to cope with their challenging situations. Other researchers revealed in their meta-analysis that emotion-focused strategies are usually not as effective as problem-focused strategies when evaluating health outcomes (Penley, Tomaka, & Wiebe, 2002). Problem-focused strategy tends to lessen or eliminate the cause of the problem. However, the majority of the adolescent women in this study narrated personal resolutions which were more emotion-focused strategies which helped them to reduce the negative emotional responses connected with their challenges but did not eliminate the cause of their problems.

## Limitations

The main limitation of this study is that the sample size is large, which is a constraint of the methodology used for the analysis in this study. IPA studies require small sample sizes because the main aim of IPA is attaining an in-depth account of individual experiences (Pietkiewicz & Smith, 2012). IPA aims for quality rather than quantity of data, thus enabling insightful analysis to be developed (Smith et al., 2009). However, the researcher applied triangulation by using both IDIs and FGDs as a method of data collection to develop a comprehensive understanding of the phenomenon under study. Using triangulation also enhanced the trustworthiness of the results, hence the use of a larger sample size than is required in IPA.

## Conclusion

Adolescent pregnancy and early motherhood are equally difficult experiences for adolescents in Ghana. This research shows that the adolescent girls felt very disappointed when they realized they were pregnant. Due to feelings of shame and stigma, some of them opted for abortions, which are mostly not professionally done. A lack of appropriate interventions such as counselling and social support for the adolescent women did not encourage them to make positive decisions such as going back to school after childbirth. Also, the males responsible for the pregnancy mostly did not take full responsibility in terms of financial support and bringing up the child. Service providers, such as teachers, sometimes failed to create a conducive environment for the adolescent women to freely access facilities without stigma.
